# The Effect of Salvianolic Acid on Vascular Protection and Possible Mechanisms

**DOI:** 10.1155/2020/5472096

**Published:** 2020-09-25

**Authors:** Yalan Wu, Suowen Xu, Xiao Yu Tian

**Affiliations:** ^1^School of Biomedical Sciences, Institute of Vascular Medicine, Chinese University of Hong Kong, Shatin, N.T. Hong Kong SAR, China; ^2^Department of Endocrinology, First Affiliated Hospital, Division of Life Sciences and Medicine, University of Science and Technology of China (USTC), Hefei, China

## Abstract

Salvia miltiorrhiza (Danshen), as an important traditional Chinese medicinal plant, has been used in China for the treatment of cardiovascular diseases for hundreds of years. Salvianolic acids (salvianolic acid A and salvianolic acid B) as the most abundant water-soluble component extracted from Salvia miltiorrhiza have attracted more and more attention from cardiovascular scientists due to its comprehensive cardiovascular actions. *In vivo* and *in vitro* studies have rendered salvianolic acid an excellent drug candidate for the treatment and prevention of cardiovascular diseases. In this review, we surveyed the protective effects of salvianolic acid A and salvianolic acid B against cardiovascular diseases and the pharmacological basis, providing a strong scientific rationale for elucidating the important role of Salvia miltiorrhiza in cardiovascular therapy. More importantly, we also hope to provide new inspiration and perspectives on the development and innovation of small-molecule cardiovascular drugs based on salvianolic acid.

## 1. Introduction

Cardiovascular disease, as one of the major noncommunicable chronic diseases that threaten human health and affect quality of life around the globe, has increasing incidence nowadays, with high rates of disability and mortality rate [1–3]. Therefore, searching for effective drugs to intervene cardiovascular diseases has become a top priority for researchers.

Chinese herbal medicine is a valuable resource to identify new small-molecule drugs against human disease. Empirical evidences from the application of either single or multiple medicinal products (including plants, minerals, or animal products) against cardiovascular disease, by Chinese medicine (TCM) practitioners, provide a lot of insights to identify potential bioactive extracts [4]. In recent years, Chinese TCM practitioners and clinicians have also integrated TCM with modern medicine, resulting in substantial improvement to dealing with cardiovascular and cerebrovascular diseases, with significantly reduced mortality and much better quality of life [4]. One of the most successful examples is the identification of several herbs or herbal extracts, as effective drugs to treat cardiovascular disease, which “improves blood circulation,” based on the TCM theory and practice to treatment of “blood stasis.” Among them include the most commonly used TCM herbs, such as Danshen (Salvia miltiorrhiza, also called red sage), Chuanxiong (Ligusticum striatum, also called Szechuan lovage), and Baishao (Paeonia lactiflora, also called white peony), which have been studied most extensively in practice, resulting in the discovery of several small molecules including salvianolic acid from Danshen [5].

Danshen belongs to the TCM herb category of promoting blood circulation and removing blood stasis. In TCM practice, it has been applied usually in combination with other herbs for thousands of years in Asian countries to treat vascular disorder-related diseases, including coronary heart disease, myocardial infarction (MI), angina pectoris, and atherosclerosis [5–8]. The effectiveness and wide usage of Danshen have led to the development of a compound Danshen dripping pill, or Dantonic®, a major innovation in TCM. Dantonic®, which has been used since the 1990s, is also the first compound TCM drug to pass the second phase of human clinical trial of the US Food and Drug Administration (FDA). A meta-analysis of 60 randomized clinical trials showed that Danshen dripping pills composed of Salvia miltiorrhiza, Panax notoginseng, and camphor have a more obvious effect than isosorbide dinitrate [5, 9, 10].

**Figure 1:**
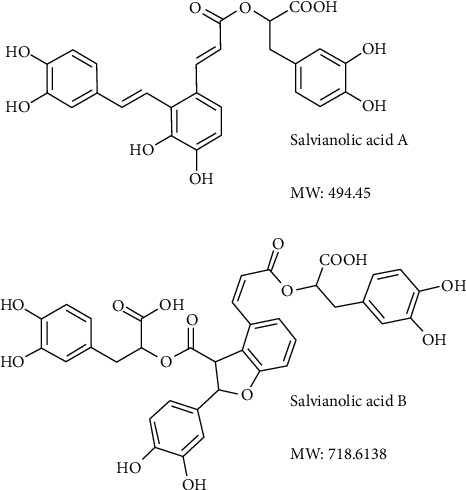
Chemical structures of salvianolic acid A (SalA) and salvianolic acid B (SalB).

So far, several chemical components of Danshen have been identified, including more than 30 lipophilic compounds with a diterpene structure, such as tanshinones I-VI, cryptotanshinone, isothiophenes I-II, and salvianphenol A, and more than 50 hydrophilic compounds, such as danshensu, salvianolic acid A (SalA), salvianolic acid B (SalB), and protocatechuic aldehyde, mostly composed of phenolic acids [5, 9, 10] (Figure 1). The contents of chemicals in S. miltiorrhiza roots vary among aqueous extracts, organic extracts, oral pills, and injections derived from the single herb of S. miltiorrhiza roots or herbal combination, like Fufang (multiple TCM herbs) prescription in China. Salvianolic acids are the most abundant water-soluble substances in Danshen. Many *in vivo* and *in vitro* studies have shown that salvianolic acid regulates multiple signal transduction pathways in vascular endothelial cells, vascular smooth muscle cells, and cardiomyocytes. In this review, we systematically surveyed the research progress of the protective effect of salvianolic acids (both SalA and SalB) on blood vessels and vascular cells, with a focus on its regulation of vascular endothelium and smooth muscle and signaling pathways related to oxidative stress, aiming to provide a scientific basis for understanding the traditional use of Salvia miltiorrhiza in vascular protection.

## 2. Evidence-Based Therapeutic Applications of Sal-Containing TCM Drugs

One of the most commonly used TCM drug Dantonic (or Fufang Danshen) has been recorded in the “*Chinese Pharmacopoeia*” (2015) which documented its application in conditions such as coronary atherosclerosis, angina pectoris, hyperlipidemia, and Alzheimer's disease in China and some other Asian countries. It is a China Food and Drug Administration- (CFDA-) approved drug since 2008. Dantonic and other forms of Fufang Danshen contain S. miltiorrhiza, Panax notoginseng, and Ligusticum wallichii, from which the composition of these drugs is originally based on TCM theory of allowing the three herbs to synergize and thus exert the desirable effects on treating cardiovascular disease and at the same time to antagonize the side effects of each other. This formulation contains many active ingredients including tanshinone, salvianolic acid, Panax notoginseng saponins, ginsenosides Rb1 and Rg1, and borneol. Many studies have proved its effectiveness, including relieving pain, promoting blood circulation, improving blood flow, reducing blood lipids, protecting blood vessels and myocardium, and improving cardiac function.

The clinical effect of S. miltiorrhiza, or its active ingredient tanshinone IIA, or Fufang Danshen, has been examined in patients with various conditions of cardiovascular diseases in several clinical studies from about 1980s. However, due to the limited information available only for literature written in Chinese, these evidences are not discussed here, although, collectively, they suggested the effectiveness of S. miltiorrhiza in coronary heart disease, stroke, thrombosis, etc. A later study showed that compared with routine care as a control, S. miltiorrhiza preparation plus routine care in patients with acute myocardial infarction reduced the mortality rate by approximately half [11]. However, many studies examined either Fufang Danshen, the extract of S. miltiorrhiza, or the compound tanshinone IIA, whereas the effects of SalA and SalB were much less explored in human subjects. For example, one study showed that Fufang Danshen reduced systolic blood pressure compared to placebo in patients with hypertension [12]. Another study showed that S. miltiorrhiza improved the prognosis of patients after percutaneous coronary intervention [13]. A meta-analysis in 2016 summarized data from over 2000 patients and concluded that S. miltiorrhiza was effective for unstable angina pectoris [14]. A later study demonstrated that S. miltiorrhiza injection reduces oxidative stress during the percutaneous coronary intervention perioperative period, increases myocardial perfusion, and promotes the recovery of cardiac function [15]. Despite all these clinical evidences, whether SalA or SalB only has effect which is similar to the total extract of S. miltiorrhiza in treating cardiovascular disease in humans is still unclear.

## 3. Protective Effects of Salvianolic Acids A and B against Oxidative Stress

Numerous studies have shown that different types of polyphenols have a wide range of pharmacological activities in endothelial cells and cardiomyocytes [16, 17]. The effect of SalA to inhibit oxidative stress implicates its potential to treat many conditions associated with excessive oxidative stress, including endothelial dysfunction, pulmonary arterial hypertension, and cardiac fibrosis [18–23], mainly through a direct effect of SalA to scavenge various types of free radicals. For example, SalA can chelate Cu^2+^ and inhibit Cu^2+^-mediated oxidation of low-density lipoprotein, reducing the production of malondialdehyde (MDA), the end product of lipid oxidation in a cell-free system [24]. The protective effects of SalA on low-density lipoprotein (LDL) oxidation implicate its potential effects on the formation of oxidized LDL (oxLDL) and consequent oxLDL-mediated proatherogenic events in the pathogenesis of atherosclerosis.

### 3.1. Direct Effect of SalA and SalB to Scavenge Free Radicals

As a polyphenol, SalA has a direct free radical scavenging effect [25]. Apart from the effect on lipid peroxidation [24], both SalB and SalA also show direct free radical scavenging ability in a cell-free system [26, 27]. Liu et al. reported that seven phenolic compounds isolated from Danshen inhibited lipid peroxidation of rat liver microsomes induced by iron/cysteine and vitamin C/NADPH [28]. These compounds also inhibit H_2_O_2_^−^-induced hemolysis of rat red blood cells in vitro [28]. SalA is generally considered a more effective antioxidant than SalB, although SalB is more abundant in Danshen [27–29]. The scavenging activity of SalB is higher than vitamin C on scavenging HO^·^, O_2_^·–^, DPPH free radicals, and ABTS free radicals, whereas its effect to chelate iron or scavenge H_2_O_2_ is lower than that of vitamin C [26]. It is reported that treatment with 5% water-soluble extract of Danshen which contained SalB for 12 weeks lowers blood cholesterol and reduces atherosclerotic plaque formation in diet-induced hypercholesterolemic rabbits, which is associated with its reactive oxygen species (ROS) scavenging capacity [30]. Moreover, SalB-treated LDL has vitamin E binding capacity and is resistant to Cu^2+^-induced oxidation [30]. Intravenous injection of SalA in a range of 0.3-3 mg/kg significantly attenuates isoproterenol-induced cardiac dysfunction and myocardial injury and improves mitochondrial respiratory function in rats with isoproterenol-induced myocardial infarction [31], which is also associated with the antioxidant capacity of SalA.

### 3.2. Modulation of Oxidative and Antioxidative Mechanisms and Molecules by SalA and SalB

SalA also exert antioxidative effects through enhancing the antioxidative arm or inhibiting oxidative enzymes in the vascular cells. For instance, SalA downregulates the expression of NADPH oxidase 4 (NOX4) [32], which produces reactive oxygen species in the disease model of pulmonary fibrosis. In addition, SalA can also upregulate the Nrf2/heme oxygenase-1 (HO-1) signaling pathway to exert its antioxidant effects [33–35]. Oxidative stress is a critical player during the process of endothelial-mesenchymal transition (EndMT) [26, 36], a cellular process important for vascular remodeling changes. Chen et al. previously reported the effect of SalA during EndMT of human pulmonary artery endothelial cells (HPAECs), as well as *in vivo* on pulmonary vascular remodeling induced by monocrotaline in rats [37]. EndMT is induced by TGF*β*1 in human pulmonary artery endothelial cells (HPAECs). SalA attenuates EndMT in HPAECs induced by TGF*β*1, through inhibition of cell migration and reactive oxygen species (ROS) production. In the monocrotaline-induced pulmonary hypertension (PAH) model, SalA improves vascular function, decreases TGF*β*1 expression, and inhibits inflammation. They also showed that these effects are attributed to SalA-induced Nrf2 translocation and subsequent upregulation of HO-1 [37]. Targeting reactive oxygen species (ROS) through Nrf2 and HO-1 seems to be one of the major downstream effectors for the effect of SalA in the vasculature.

Excessive ROS production enhances the proliferation of aortic vascular smooth muscle cells (VSMCs). Hung et al. showed that SalA and SalB inhibit the proliferation of rat aortic VSMC cell line A10 which is stimulated by homocysteine (a cardiovascular risk factor known to trigger ROS). By using proteomics, they further showed that Salvia miltiorrhiza root aqueous extract containing 3,4-dihydroxybenzoic acid, 3,4-dihydroxyphenyl lactic acid, and salvianolic acid B inhibits the activation of protein kinase C and p44/42 MAP kinase to reduce ROS induced by homocysteine in VSMCs [38]. Interestingly, the aqueous extract also reduces carbonylation (a protein modification induced by oxidative stress) of several cytoskeletal and chaperone proteins (such as vimentin, a4 tropomyosin, and GRP75) in VSMCs [38]. ROS scavenging activity of salvianolic acid also mediates its protective effect on cardiomyocytes against doxorubicin-induced cardiotoxicity, through SalA-induced transition of HO^·^ produced by electron transfer on doxorubicin cysteine free radicals to H_2_O_2_ as the end product [39]. Treatment with salvianolic acid, containing mostly SalB (64.92%), has similar effect against doxorubicin-induced cardiotoxicity by inhibiting oxidative stress [40]. These studies suggest that SalA and SalB have potent antioxidant effects either by direct scavenging or by modulating expression of oxidant/antioxidant enzymes and upstream regulators including protein kinases.

Similar to its effect of directly reacting with free radicals, SalA and SalB also directly interact with phosphoproteins, many of which are important players in signal transduction. For instance, Liu et al. studied the putative protein targets of salvianolic acid [41] and showed that salvianolic acid binds to many proteins including an important vascular extracellular matrix protein matrix metalloproteinase MMP-9 [42], as well as some other protein targets including extracellular-signal-regulated kinase 1/2 (ERK1/2), c-Jun NH(2)-terminal kinase (JNK), and p38 mitogen-activated protein kinase pathway [43–48], most likely through the interaction of SalA and SalB with the phosphotyrosine or phosphoserine/threonine binding domains [49, 50]. Using a similar approach, Sperl et al. also reported that SalA and SalB are inhibitors of protein-protein interactions at the SH2 domain of the Src family kinases Src and Lck. The potency of SalA and SalB (from 0 to 100 *μ*mol/L) in combination with Src and Lck is higher than that of rosmarinic acid, a known Lck SH2 domain inhibitor [51]. Since Lck is a T cell-restricted Src family protein tyrosine kinase and is critical in the TCR-mediated signaling pathway, the activity of Lck SH2 domain makes SalA and SalB optimal candidates for immune-suppressive anticancer agents, similar to rosmarinic acid [52, 53]. A recent report by Wang et al. used an ELISA-like high-throughput screening assay to show that SalB and rosmarinic acid have high affinity to bind and to inhibit SH2 domain of CD36, a membrane receptor of oxLDL, thus preventing oxLDL from being taken up by macrophages [54]. Since both SalA and SalB have a similar core structure of rosmarinic acid [51], their high affinity for the Src and the SH2 domain of CD36 indicates the role of immunomodulators in the cardiovascular protection of salvianolic acid. The interaction with CD36 might also have important implications in plaque cell formation during atherosclerosis.

## 4. Salvianolic Acid A

The beneficial effects of SalA have been studied in the cardiovascular system, including antioxidative, antithrombotic, antifibrotic, anti-inflammatory, and pleotropic effects to protect the myocardium and macro- and microvasculature [55].

### 4.1. Effects of SalA on Vascular Dysfunction

Endothelial dysfunction is the initiating event leading to vascular inflammation, vascular remodeling, and eventually causing cardiovascular diseases. Therefore, there is a continuous search for new drugs and new drug targets against endothelial dysfunction. Several studies explored the effect of salvianolic acids on endothelial dysfunction using various models. Sun and others [19, 56, 57] studied the effects of SalA on endothelial dysfunction and vascular remodeling using disease models including angioplasty-related restenosis, injury-induced neointimal hyperplasia, oxidative stress, and advanced glycation end product- (AGE-) induced endothelial dysfunction through enhancing endothelial nitric oxide synthase (eNOS) and its upstream regulators such as cAMP-related signaling. Studies also demonstrated that SalA improves endothelium-dependent vasodilation impaired in both models of spontaneously hypertensive rats [22] and the high-fat, high-fructose-induced diabetic rats [57], through enhancing nitric oxide (NO) bioavailability, without affecting blood pressure. SalA can also reverse ischemia/reperfusion-induced reduction in NO bioavailability by reducing MKP-3 (mitogen-activated protein kinase phosphatase 3) in HUVECs [58].

Apart from NO-related pathways, SalA also regulates many other targets which might indirectly modulate NO bioavailability. For instance, SalA inhibits endothelial cell damage induced by AGE through enhancing the antioxidant capacity and the upregulation of eNOS [57]. A recent study showed that SalA is a safe endothelin-1 type A receptor (ETAR) antagonist (IC50 = 5.7 *μ*mol/L) in HEK293 cells overexpressing ETAR [59], suggesting that SalA may be effective in treating hypertension because endothelin-1 is a potent vasoconstrictor. SalA reduces angiotensin II-induced proliferation of human endothelial cell by inhibiting ROS production and blocking phosphorylation of Src and Akt [33]. SalA also attenuates PDGF-BB- (platelet-derived growth factor-BB-) induced VSMC proliferation and migration by inhibiting PDGFR*β*/ERK [56] and the cyclic adenosine monophosphate/protein kinase A/CREB signaling, thus preventing neointimal hyperplasia in rat [19].

In addition to the effect on large vessels, SalA also maintains endothelial homeostasis in microvascular circulation. Teng et al. demonstrated that in SHRs, SalA treatment between 2.5 and 10 mg/kg/day for 4 weeks attenuates the retinal microvascular inward remodeling and improves the microvascular function of the mesentery *in vivo* [22]. They also showed that SalA improves endothelial monolayer integrity *in vitro*, suggesting the therapeutic potential of SalA to prevent organ damage caused by vascular remodeling in the context of hypertension [22]. Yang et al. showed that SalA improves endothelial homeostasis and reduces permeability by modulating tight junction protein ZO-1 and actin cytoskeletal reorganization and reducing ROS in HUVECs exposed to ischemic reperfusion injury. Such effect is mediated through the inhibition or inactivation of p38 MAPK and downregulation of VLDL receptor in endothelial cells [60]. Both studies showed that SalA helps to maintain endothelial homeostasis in microcirculation [22].

### 4.2. Effects of SalA on Vascular Inflammation

Dysregulated endothelium triggers vascular inflammatory response through interaction with immune cells, among which macrophage is an important player especially during atherogenesis. SalA inhibits lipopolysaccharide- (LPS-) induced upregulation of proinflammatory mediators by targeting IKK*β* (NF-*κ*B kinase inhibitor) and the antioxidative enzyme HO-1, both inhibiting NF-*κ*B and its target genes including COX-2, iNOS, TNF-*α*, and IL-6 in macrophages [61, 62]. SalA also attenuates angiotensin II-induced macrophage apoptosis by inhibiting Akt and NF-*κ*B activation, both of which are involved in response to various proinflammatory stimuli [63]. It remains to be investigated whether SalA affects cholesterol uptake and efflux, the subsequent foam cell formation, and efferocytosis of macrophages, which are also important functions of plaque macrophages contributing to the enlargement and resolution of atherosclerotic plaque. Moreover, acetyl-modified SalA inhibits platelet aggregation induced by various proagglutination stimulations, including thrombin, collagen, ADP, and arachidonic acid, suggesting that acetyl-SalA has more potent antithrombotic activity than SalA. Subsequent *in vitro* and *in vivo* studies confirmed that SalA inhibits ADP and collagen-induced platelet aggregation and arterial thrombosis in mice [64–67]. These evidences indicated that SalA acts on several vascular cell types through similar signaling molecules including NF-*κ*B and HO-1 to inhibit inflammatory responses.

## 5. Salvianolic Acid B

### 5.1. Effects of SalB on Vascular Dysfunction

Similar to SalA, SalB can also act on the Nrf2-ARE signaling pathway and the p38-MAPK signaling pathway, both of which are closely associated with atherogenesis. Lee et al. showed that SalB inhibits VSMC proliferation and migration induced by PDGFs, accompanied by the upregulation of HO-1 to reduce ROS production [68]. Similarly in HUVECs, SalB upregulates HO-1 and, as a consequence, also inhibits the activation of NF-*κ*B during the process of cell migration and inflammation induced by TNF-*α*. Further mechanistic studies showed that the upregulation of HO-1 by SalB is Nrf2-dependent. Ling et al. examined the vascular protection of SalB in angiotensin II-induced hypertensive mice. They found that SalB (25 mg/kg/day) treatment for 11 days reverses impaired endothelial function and significantly inhibits AT1 receptor-dependent vascular oxidative stress through inhibition of AT1 receptor, as well as NADPH oxidases NOX2 and NOX4 [69]. However, compared to SalA, there have been less studies on the potential benefits of SalB against vascular dysfunction-related diseases.

### 5.2. Effects of SalB on Vascular Inflammation

SalB can act on various aspects of the interaction between endothelial cells and immune cells to dampen vascular inflammation, which is particularly important at the early stage of atherogenesis. SalB inhibits TNF-*α* induced endothelial cell-leukocyte adhesion molecule expression on human aortic endothelial cells, in particular the two major adhesion molecules VCAM-1 and ICAM-1, through inhibition of NF-*κ*B activity induced by TNF-*α*, without affecting E-selectin (another major adhesion molecule expressed on the endothelial cells triggered by inflammatory stimuli) [43]. Besides TNF-*α*, SalB also targets interferon-induced response by probably a direct inhibition with IFN-induced phosphorylation of JAK2 (at tyrosine 1007/1008) and also phosphorylation of STAT1 (at tyrosine 701 and serine 727) [70]. This leads to the downregulation of IP-10, a target chemical attractant downstream of STAT1, and suppression of cell adhesion in IFN-*γ*-stimulated endothelial cells. In addition, SalB can upregulate PIAS1 and SOCS1, two inhibitory factors of the proinflammatory JAK-STAT1 signaling pathway [70]. Recently, Yang et al. found that SalB can not only inhibit the expression of YAP/TAZ and inflammatory proteins (JNK, NF-*κ*B, and TNF-*α*) in EC and pericytes but also protect EC and pericytes from oxidative stress and apoptosis. *In vivo* experiments confirmed that SalB reduces atherosclerosis in ApoE^−/−^ mice and reduces inflammatory markers including IL-6, IL-1*β*, TNF-*α*, and oxLDL in the serum sample of ApoE^−/−^ mice fed a high-fat diet [71].

Platelet-mediated vascular inflammation also contributes to the development and progression of atherosclerosis. A growing body of evidences suggests that SalB may be a potential candidate for the treatment of various atherosclerotic diseases by acting on platelets or endothelial-platelet interaction. For instance, SalB attenuates platelet-triggered inflammation in endothelial cells mediated by NF-*κ*B activation [71–73]. In platelets, SalB inhibits both ADP and thrombin-induced platelet aggregation by reducing the release of soluble P-selectin and antagonizing the activity of phosphodiesterase and P2Y12 receptors expressed on the platelets [71–73]. NF-*κ*B inhibition by SalB is one of the major mechanisms to reduce platelet aggregation and intravascular coagulation in vascular inflammation by SalB acting on both platelets and endothelial cells [72–77].

Effects of SalB on inflammatory response of VSMCs have also been studied. Studies showed that SalB inhibits ERK1/2 and JNK phosphorylation in VSMCs and reduces COX-2-mediated production of PGE_2_, a major inflammatory prostanoid, and NADPH oxidase activity, therefore reducing the proliferation and migration of VSMCs through suppression of MMP-2 and MMP-9, two major enzymes responsible for degradation of the adventitial extracellular matrix, and stimulation of smooth muscle cell migration and proliferation [46, 47]. As a result, in ApoE knockout mice fed with a high-cholesterol diet, supplementation with 0.3% of SalB for 3 months protects mice from atherosclerosis by reducing vascular intimal thickness, accompanied by decreasing COX-2, MMP-2, and MMP-9 expression [46, 47]. In addition, SalB also inhibits TNF-*α*, angiotensin II, or H_2_O_2_-induced MMP-2 activation in VSMCs through the inhibition of NADPH oxidase-independent ROS generation [48].

In addition to its effect on endothelial cells against inflammatory responses, SalB also modulates the immune cell population in the plaque. For instance, SalB can retard the maturation of dendritic cells from monocytes accelerated by oxLDL through inhibition of costimulatory molecules CD40 and CD86, mediators of antigen presentation including CD1a and HLA-DR, and proinflammatory and immunomodulatory cytokine IL-12, IL-10, and TNF-*α* production [78]. Importantly, SalB also activates PPAR*γ* to attenuate oxLDL-induced TLR4 and MyD88 innate immune pathway and its downstream p38-MAPK signaling cascade in dendritic cells [78]. In macrophages, activation of PPAR*γ* along with LXR*α* by SalB promotes cholesterol efflux through ABCA1 upregulation and reduces LDL uptake by antagonizing CD36-mediated lipid uptake in monocyte-derived macrophages [79], thereby preventing macrophages to become a foam cell [54, 79, 80]. In microglia cells, SalB also reduces the production of NO, TNF-*α*, IL-1b, and ROS induced by LPS through similar mechanisms as those found in endothelial cells [81]. Moreover, SalB might also regulate T lymphocytes, another major player to amplify inflammation in atherogenesis, through inhibition of IL-2, IL-4, TNF-*α*, and IFN-*γ* production, therefore decreasing T cell activation reflected by inhibition of CD25 and CD69, due to the suppression of transcriptional activity of AP-1, NF-*κ*B, and Oct-1 [82]. The effect of SalB on NF-*κ*B is related to the inhibition of JNK and degradation of I*κ*B*α*. These evidences also suggested a potential immunomodulatory effect of SalB, which is different from SalA.

## 6. Conclusions and Future Perspectives

**Figure 2:**
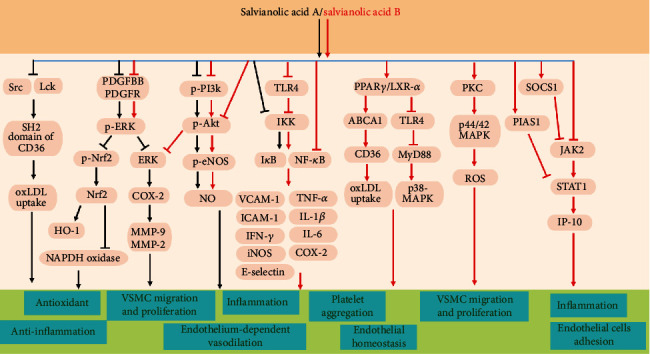
The major signaling pathways involved in the vascular protection of salvianolic acid A (SalA) and salvianolic acid B (SalB).

As major bioactive components from Salvia miltiorrhiza (Danshen), there are still many ongoing developments to studying the effect of SalA and SalB on targeting different tissues and various signaling pathways to explore their effect on human diseases, not only restrained to cardiovascular diseases (summarized in Figure 2). Improving the formulation techniques to preserve the bioactive property and stability in vivo is still needed. Extensive evidences have demonstrated the effectiveness of SalA and SalB on vascular dysfunction and vascular inflammation using many animal models to mimic human cardiovascular disease. Both SalA and SalB have effects against oxidative stress, platelet aggregation, coagulation, thrombosis, endothelial dysfunction, and inflammation targeting multiple vascular cell types. As a potent polyphenol, SalA and SalB have direct ROS scavenging ability against different types of free radicals. A more quantitative and reliable method to directly measure scavenging property against free radicals such as superoxide, H_2_O_2_, or biomolecules such as oxLDL in both cell-free and in vivo situation will be extremely informative for future drug development and pharmacokinetic study, for example, using electron spin resonance spin trapping technique. To conclude, the prominent antioxidant, anti-inflammatory, and antifibrotic effects of SalA and SalB translate into the broad cardiovascular actions of Danshen and implicate that both bioactive compounds represent viable drug candidates in cardiovascular disease therapy.

In addition, SalA and SalB might have direct effect to regulate protein phosphorylation on kinases such as ERK, MAPK, and SH2 domain of the Src family kinases. More mechanistic study, for example, using nonbiased proteomics to detect protein phosphorylation treated with SalA and SalB, will provide more information on their interactions with intracellular signaling molecules, in the purpose of detecting new targets. Further animal study using transgenic animals to examine the effect of SalA and SalB in disease models will also be helpful.

## Data Availability

The data used to support the content of this manuscript are available upon request to the corresponding author.
